# Temporal and technical variability of human gut metagenomes

**DOI:** 10.1186/s13059-015-0639-8

**Published:** 2015-04-08

**Authors:** Anita Y Voigt, Paul I Costea, Jens Roat Kultima, Simone S Li, Georg Zeller, Shinichi Sunagawa, Peer Bork

**Affiliations:** Structural and Computational Biology Unit, European Molecular Biology Laboratory, 69117 Heidelberg, Germany; Department of Applied Tumor Biology, Institute of Pathology, University Hospital Heidelberg, 69120 Heidelberg, Germany; Molecular Medicine Partnership Unit (MMPU), University of Heidelberg and European Molecular Biology Laboratory, 69120 Heidelberg, Germany; School of Biotechnology and Biomolecular Sciences, University of New South Wales, 2052 Sydney, Australia; Max Delbrück Centre for Molecular Medicine, 13125 Berlin, Germany

## Abstract

**Background:**

Metagenomics has become a prominent approach for exploring the role of the gut microbiota in human health. However, the temporal variability of the healthy gut microbiome has not yet been studied in depth using metagenomics and little is known about the effects of different sampling and preservation approaches. We performed metagenomic analysis on fecal samples from seven subjects collected over a period of up to two years to investigate temporal variability and assess preservation-induced variation, specifically, fresh frozen compared to RNALater. We also monitored short-term disturbances caused by antibiotic treatment and bowel cleansing in one subject.

**Results:**

We find that the human gut microbiome is temporally stable and highly personalized at both taxonomic and functional levels. Over multiple time points, samples from the same subject clustered together, even in the context of a large dataset of 888 European and American fecal metagenomes. One exception was observed in an antibiotic intervention case where, more than one year after the treatment, samples did not resemble the pre-treatment state. Clustering was not affected by the preservation method. No species differed significantly in abundance, and only 0.36% of gene families were differentially abundant between preservation methods.

**Conclusions:**

Technical variability is small compared to the temporal variability of an unperturbed gut microbiome, which in turn is much smaller than the observed between-subject variability. Thus, short-term preservation of fecal samples in RNALater is an appropriate and cost-effective alternative to freezing of fecal samples for metagenomic studies.

**Electronic supplementary material:**

The online version of this article (doi:10.1186/s13059-015-0639-8) contains supplementary material, which is available to authorized users.

## Background

Microbial communities that inhabit the human gut are essential to human health. To better understand the role of gut microbes in health, major efforts have been undertaken including large-scale studies such as the European Metagenomics of the Human Intestinal Tract (MetaHIT) project and the US American Human Microbiome Project (HMP) [1,2]. These studies have provided insights into the gut microbial community composition in healthy human individuals. Changes in the microbial community composition have been associated with diet [3,4] as well as with multiple diseases, such as atherosclerosis, inflammatory bowel diseases and obesity [5-7].

In addition to these cross-sectional studies that compared healthy and diseased cohorts, longitudinal studies have helped shed light not only on the community compositional variability but also on the temporal variability, providing a more complete picture of the factors that shape the gut microbiome in health and disease. Several studies have demonstrated considerable between-subject variability of the gut microbial composition. However, the gut microbiome has been described to be constrained around a highly personal and stable composition within each healthy subject over time [8-12].

Perturbation of the human gut microbiome is known to occur as a result of antibiotics treatment, a frequently prescribed medication. Antibiotic intervention leads to a rapid decrease of diversity and post-treatment recovery is slow and incomplete, even up to 4 years after the treatment [13-17]. Resistant bacterial species, as a result of antibiotics treatment, can persist over years [18-20] and the resistance potential of gut microbiota displays regional differences [21,22]. Similarly, there are indications of other long-term community shifts caused by endogenous (for example, disease) or environmental perturbations (for example, diet and lifestyle change [3,4,23]) that have not yet been studied in depth.

Studies on the temporal variability of the gut microbiome have mostly been performed over short periods (weeks to one year; for example, [8,12,23,24]) and only rarely over long periods (5 and 12 years [9,11]). The methods of deriving the taxonomic community composition were primarily based on PCR-denaturing gradient gel electrophoresis (PCR-DGGE; for example, [25]), 16S rRNA gene sequencing (for example, [26,27]), and the HITChip microarray (for example, [11,28]). Only two studies [12,29] have so far analyzed longitudinal non-amplified metagenomic shotgun sequencing data that were collected from 43 subjects in the context of the HMP [1]. However, the majority (41 out of 43) were only sampled twice, making it difficult to assess temporal stability.

Despite their common aim to better understand microbial community shifts over time, the aforementioned studies do not attempt to quantify different sources of variability, from technical to biological ones. In particular, technical aspects have been shown to be important for the comparison between data sets. Limited comparability in human microbiome data sets often results from differences in sample preservation and DNA isolation protocols as well as readout methods (for example, sequencing of different 16S rRNA gene regions or application of different sequencing technologies). A meta-analysis [30] assessing the effect size of technical differences on data comparability showed that samples rather cluster by study or the methods applied (for example, for DNA isolation) than by the parameter of interest (for example, disease state). To counteract these batch effects, the International Human Microbiome Standards (IHMS) project was launched to suggest standards for sample processing (mainly DNA isolation) with the goal to maximize future data comparability. However, different storage conditions of a fecal sample can also impact the compositional readout, as different microbes respond differently to environmental exposure [31]. Research in this direction has been conducted previously to compare different storage and preservation conditions (for example, different temperatures or preservatives such as RNALater) [32,33]. RNALater, a quaternary ammonium salts-based solution, is commonly used as a logistically convenient solution to preserve RNA from biological samples at room temperature when freezing is not possible, and was recently also considered for omics technologies [34]. It was shown to have a minor effect on the recovered composition and thus represents a potential alternative to immediate freezing [35-37]. To date, the technical variability on a taxonomic and functional level has not been put in the context of temporal and within-sample variability (meaning within the stool from a single bowel movement).

We collected fecal samples over up to two years from seven subjects to investigate the temporal variability and individuality of the human gut microbiome using metagenomic shotgun sequencing. To disentangle technical, temporal and between-subject variability we contrasted the variability of microbial community composition within a fecal sample [38,39] with the variability introduced by different preservation methods, RNALater or freezing after two different time intervals. By comparing the fecal metagenomes of the seven subjects over time and in the context of 888 published metagenomes, we generally found between-subject variability to be much larger than within-subject variability. This high degree of individuality can, however, be disrupted by antibiotic treatment, which in one subject triggered a large and long-lasting community shift. Bowel cleanse was also investigated but did not appear to cause a major disturbance. Technical variability (within-sample and preservation-induced variability) was smaller than temporal within-subject variability and therefore we propose RNALater as an alternative to fresh freezing fecal samples.

## Results and discussion

### Study design

**Figure 1:**
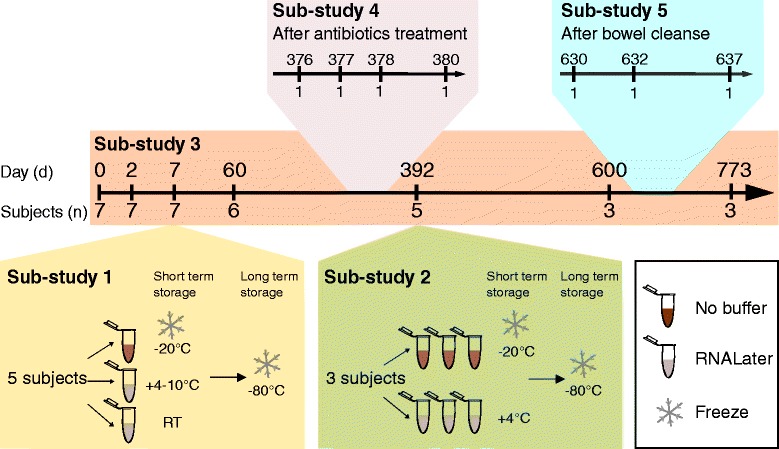
Overview of the study design. This variability assessment was subdivided into five sub-studies. The comparison of technical variability due to fecal preservation methods and stool homogeneity was addressed in sub-studies 1 and 2, respectively. The conditions for the different preservation methods and the numbers of replicates taken are described for these sub-studies. Sub-study 3 was about the temporal variability within and between subjects. One subject underwent antibiotics treatment (sub-study 4) and bowel cleanse (sub-study 5) in the time course of the study. The sampling time points and number of subjects that collected fecal samples are indicated in the timeline.

Fecal samples were self-collected from seven adults at short (few days) and longer (weeks to months) time intervals (Additional file 1). All subjects were considered healthy at the time of sampling, unless stated otherwise (see Material and methods). The study was split into five sub-studies as shown in Figure 1. Out of the seven subjects, five subjects performed sampling for more than one year while three subjects collected over more than two years (sub-study 3). At two time points, seven days (d_7_; sub-study 1) and 392 days (d_392_; sub-study 2) after the first sampling event, feces from three and five subjects, respectively, were collected and replicates either frozen or preserved in RNALater. One subject (*Alien*) collected additional fecal samples after antibiotics treatment (d_376–380_, sub-study 4) and bowel cleanse (d_630–637_, sub-study 5).

All fecal samples were subjected to whole genome shotgun sequencing and the data analyzed at species-level using mOTUs (metagenomic operational taxonomic units based on single-copy phylogenetic marker genes [29]), and at a number of functional levels: clusters of orthologous groups (COGs) [40], KEGG (Kyoto Encyclopedia of Genes and Genomes) groups of orthologous genes (KOs), modules and pathways [41].

### Preservation-induced variability of the fecal species community

Biological samples are generally stored frozen or processed immediately to maintain their integrity. However, this is often logistically inconvenient, especially in remote areas. In contrast, preservation in RNALater eliminates the need for immediate freezing or sample processing. RNALater is an aqueous solution that preserves biological samples by protecting especially RNA from degradation (for example, [42]). The solution penetrates and stabilizes the sample for later analysis. According to the manufacturer’s instruction, these samples are stable at room temperature (RT) for up to one week, at +4°C for one month and at −20°C and −80°C indefinitely. Thus, the usage of RNALater for sample collection would facilitate sample preservation and shipping prior to metagenomic analysis.

We collected fresh samples from which aliquots were frozen immediately (to be used as reference samples) or preserved in RNALater. At d_7_, RNALater-preserved samples from five subjects were kept at both +4 to 10°C and RT for one week. However, at d_392_ RNALater-preserved samples were kept at +4°C for 24 h from three subjects before storing at −80°C (sub-studies 1 and 2; Figure 1).

**Figure 2:**
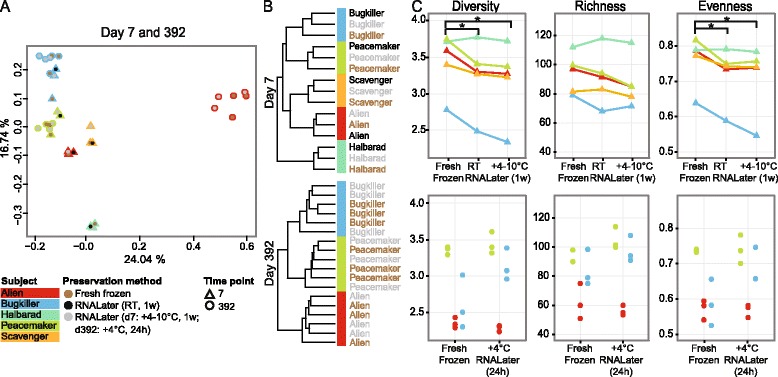
Comparison of methods for fecal sampling. **(A,B)** The samples collected in sub-studies 1 (d_7_) and 2 (d_392_) clustered by subject **(A)** and time point **(B)** but not according to the preservation method (frozen, RNALater (+4 to 10°C or RT for 1 week (1w) on d_7_; frozen and RNALater +4°C, 24 h on d_392_) applied (complete linkage clustering based on Euclidean distance). **(C)** Shannon diversity index, richness and evenness are shown for d_7_ (upper panels) and d_392_ (lower panels) and statistically significant differences are indicated by asterisks with *P*-value ≤0.05 (unpaired Wilcoxon test).

**Figure 3:**
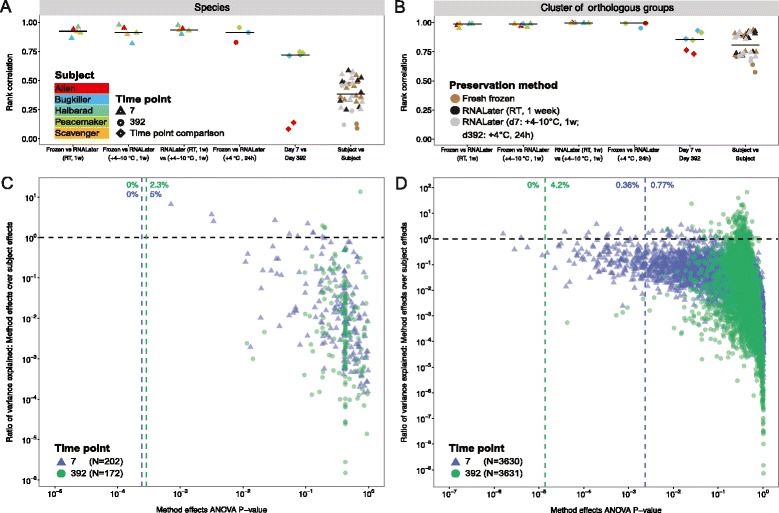
Comparison of technical, temporal within-subject and between-subject variability at taxonomic and functional levels. **(A,B)** Spearman’s rank correlations of species profiles **(A)** and cluster of orthologous groups (COG) profiles **(B)** were highly correlated between different preservation methods (frozen versus RNALater (+4 to 10°C or RT for 1 week (1w) each), RNALater at +4 to 10°C versus RNALater at RT and frozen versus RNALater at +4°C, 24 h), less correlated between sampling time points (d_7_ versus d_392_), with lower correlation seen between subjects. Black bars represent group-wise medians. Relatively lower correlation between time points was apparent for the *Alien* samples (red squares), which were taken before and after antibiotics treatment (see main text). **(C,D)** Preservation-induced changes relative to between-subject variability was quantified by two-way ANOVA for species **(C)** and COGs **(D)**, including only features with a relative abundance of at least 0.01% in three or more samples. In total, 7.3% and 5.33% of species and COGs, respectively, showed greater between-method variation than between-subject variation (features above the horizontal black line), but this was statistically significant for only 0.36% of COGs and none of the species tested (Benjamini-Hochberg false discovery rate, α = 0.05, Additional file 5). Vertical blue and green lines represent the threshold for statistical significance for d_7_ and d_392_, respectively. Percentages left and right of these lines identify the fractions of statistically significant and insignificant features with larger between-protocol variation for each time point, respectively.

To analyze the taxonomic variability of frozen and RNALater-preserved replicates, we performed hierarchical clustering of the mOTU abundances based on Euclidean distance. This analysis revealed that samples from the same subject clustered together irrespective of the preservation method. This similarity held true for both d_7_ and d_392_ with the exception of subject *Alien*, who underwent an antibiotics treatment in between these time points (Figures 2A,B and 3A). Within the cluster of each subject (at d_392_), the replicates did not cluster by the preservation protocol (Figure 2B), suggesting that biological within-sample variability was larger than preservation-induced effects.

**Figure 4:**
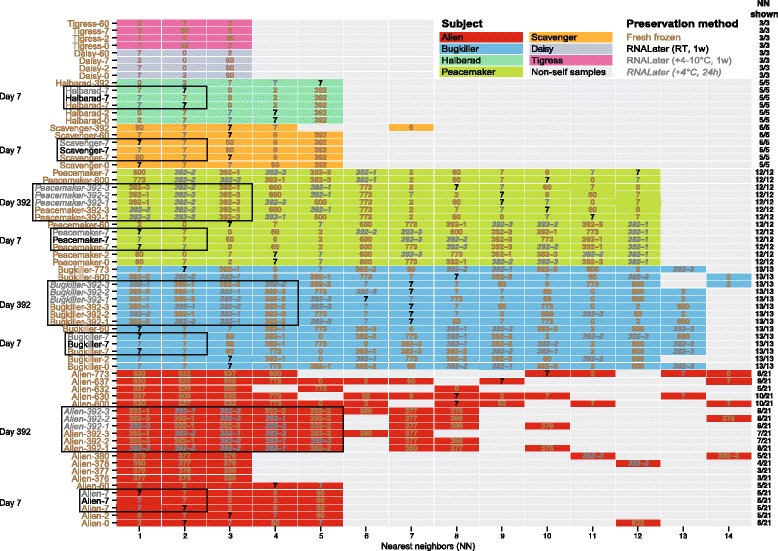
Nearest neighbor plot. The mOTU abundances of the fecal metagenomes of the time series and replicates were clustered in the context of 888 published metagenomes. Only the 14 nearest neighbors (NN) are shown for visual clarity. The colored boxes indicate the respective subject. Non-self samples (samples from another subject, including HMP and MetaHIT) are shaded in grey. Subjects are color-coded, sampling time points are indicated and text color corresponds to the preservation condition of each sample (see key). The column on the right shows how many NNs of each respective sample are depicted, indicating the subject-specificity of the clustering (complete linkage clustering based on Euclidean distances). The figure shows that, with very few exceptions, all time series samples and all fecal replicates (from d_7_ and d_392_) from one subject were closer to each other than to any other sample from another subject. Pre-treatment samples from subject *Alien* were nearest neighbors to each other while the samples right after the treatment (d_376–380_) had highest similarity to each other but not to the pre-treatment samples. The samples collected long after the treatment (d_600–773_) were most similar to each other but a slow recovery to the pre-antibiotics state was visible since pre-treatment samples are among the 14 neighbors shown.

To extend this observation, we clustered all collected samples from all subjects in the context of 888 published metagenomes from MetaHIT and HMP (Figure 4; details in Material and methods). We found that the samples from d_7_ and d_392_ had other samples from the same subject as nearest neighbors. All d_7_ samples had the other two replicates from d_7_ as nearest neighbors (Figure 4). For d_392_, the first three *Peacemaker* and four *Bugkiller* neighbors were their corresponding replicates from d_392_. For subject *Alien*, all samples from d_392_ clustered together but the nearest neighbor was not necessarily the samples preserved under the same condition. Thus, the samples did not cluster by preservation method. Taken together, these results show that RNALater does not introduce a bias in the overall microbiome composition and its effect is smaller than within-subject variability.

We repeated the taxonomic analyses using gene abundances summarized at different functional levels. Relative abundance of orthologous groups, that is, COG and KO profiles, (see Material and methods) of all collected samples were clustered in the context of 888 published metagenomes from MetaHIT and HMP (Additional files 2 and 3). For both COG and KO profiles, the nearest neighbor of samples from d_7_ and d_392_ were very similar to those seen in taxonomic clustering (Figure 4). Using COG abundances, with the exception of one *Peacemaker* sample, all d_7_ replicates clustered together, and for *Peacemaker* and *Alien* four and five of the d_392_ sample replicates, respectively, clustered together. Using KO profiles, the clustering of samples was similar.

To get a deeper insight into potential preservation-induced changes of the microbiome, we compared indices for species diversity and community evenness. At d_7_, RNALater-preserved samples (storage at RT or at +4 to 10°C) compared to the immediately frozen samples showed a significant decrease in their Shannon diversity index (*P* = 0.016 and *P* = 0.0008, unpaired Wilcoxon-test) and species evenness (*P* = 0.016 and *P* = 0.016, unpaired Wilcoxon-test) but not richness (*P* = 0.056 and *P* = 0.056, unpaired Wilcoxon-test). In contrast, at d_392_, RNALater preservation did not have the same effect on these ecological indices (Figure 2C).

To determine preservation-induced and temporal within-subject and between-subject differences, we correlated mOTU, COGs, KOs, KEGG modules and pathways (Spearman correlation) between different preservation techniques, sampling time points and subjects (Figure 3A,B; Figure S3A-C in Additional file 4). We found that the similarity between protocols is consistently high for both species and COGs (minimum Spearman’s r = 0.82 and 0.95, respectively), similar to previous findings [36]. Due to our longitudinal study design we could extend the analysis performed by Franzosa *et al.* [36], and verify that the correlation between time points was lower for both species and COGs (maximum Spearman’s r = 0.75 and 0.93, respectively) than between preservation methods. Between-subject correlations were even lower than between-time point correlations.

To estimate differences in taxonomic (species) and functional (eggNOG COGs, and KEGG KOs, modules and pathways) composition between frozen and RNALater-preserved samples from d_7_ and d_392_, we performed two-way ANOVA testing on both the taxonomic and functional relative abundances (see Material and methods). We found that 5.0% (d_7_) and 2.3% (d_392_) of the species with a relative abundance exceeding 0.01% in at least 3 of the 33 tested samples varied more between preservation methods than between subjects. However, none of these were statistically significant after correction for multiple hypothesis testing (Benjamin-Hochberg α = 0.05; Figure 3C; Additional file 5). For d_7_ and d_392_, 0.77% and 4.2% of the COGs, respectively, varied more between the preservation methods than between subjects, but only 0.36% of COGs were statistically significant (Figure 3D; Additional file 5), which is in the range of previous findings [36]. We found that 0.72%, 0% and 0% of the KOs, modules and pathways, respectively, varied more between preservation methods than between subjects (Figure S3D-F in Additional file 4).

In summary, RNALater appears, in line with a previous publication [36], to be a suitable alternative to immediate freezing at least for short-term storage of a few days, as the variability between protocol replicates is lower than that between time points of the same subject and between subjects.

### Within-sample variability of the fecal species community

It was previously shown that there is considerable spatial within-sample variation of parasites in human feces [38] and low abundant bacteria were only sporadically detected in all replicates of the same sample [39]. To address within-sample and technical reproducibility in our study, triplicates at distinct sites of the same fecal sample were collected from three subjects at d_392_ using two preservation protocols (RNALater and freezing; Figure 1, sub-study 2).

For two subjects the replicates showed only minor variation in ecological indices (Shannon diversity index, species richness and community evenness). Larger fluctuations were detected for diversity and evenness of fresh frozen samples from subject *Bugkiller* only (Figure 2C, lower panel). Nevertheless, all replicates clustered by subject (including *Bugkiller*) in the context of the samples collected on d_392_ (Figure 2B). To set within-sample variation in the context of all time series samples and the MetaHIT and HMP samples (N = 888), we clustered all samples together (Figure 4). The replicates from all three subjects had the other replicates from the same subject as nearest neighbors. All replicates from subject *Alien* clustered by d_392_ but not with the pre-/post-treatment samples, highlighting the drastic change introduced by the treatment. These results for *Alien* remained the same when clustering based on abundances of functional categories (Additional files 2 and 3). This implies that subject-specificity and community similarity is high for all replicates of a fecal sample with only minor fluctuations in diversity and evenness. Together with the fact that replicates preserved under different conditions did not cluster by preservation method (Figures 2B and 4) this supports our study design which was based on samples that were deliberately not homogenized before aliquoting since (i) samples included in our study were self-collected by lay participants and usually not homogenized in large metagenomic studies and (ii) we aimed to assess within-sample variability.

### Temporal variability of fecal microbial communities

**Figure 5:**
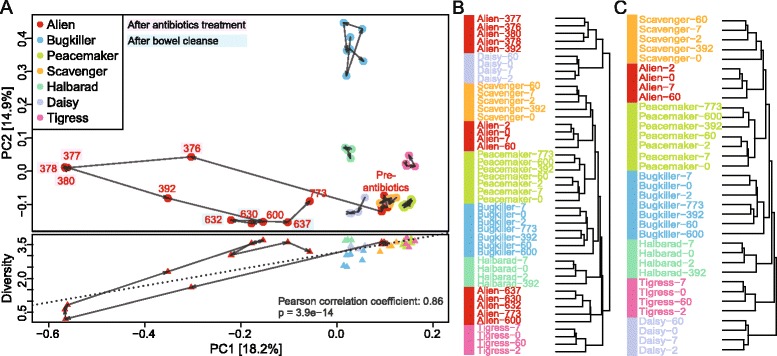
Clustering of the complete time series data set. **(A)** The unperturbed microbiomes of the subjects were stable except for the *Alien* samples, which showed a decline of the Shannon diversity index upon antibiotics treatment (d_376–392_) while the bowel cleanse (d_630–637_) had no detectable effect. The separation of the post-antibiotics from the pre-antibiotics samples along the first principal coordinate (PC1) based on Jensen-Shannon divergence distances was significantly correlated with the decline of the Shannon diversity index (dotted line, *P*-value = 3.9e^−14^) (**A**, lower panel) and explained the separate clustering of pre-and post-treatment samples (**A**, upper panel and **(B)**). **(C)** The unperturbed gut microbiome was highly personal and when omitting the post-antibiotics samples, all subjects that collected time series samples over up to two years can be resolved.

In order to assess how technical variability compares to temporal variability, all samples collected by the seven subjects were clustered. It showed that the temporal variability was small and the samples clustered by subject except for *Alien* (Figure 5A,B). Omitting all samples taken from *Alien* after antibiotics treatment resulted in consistent clustering by subject (Figure 5C), showing high subject-specificity and individuality of the gut microbiome. In order to test whether the individuality of the gut microbiome persists on the background of 888 published metagenomes from MetaHIT and HMP, we clustered them together and show the nearest neighbors in Figure 4. The time series samples from the seven subjects were closest to other samples from the same subject rather than to another subject. This was also seen for the 43 subjects in the HMP study, which have multiple time-points. Only few samples from our dataset had the sample of another subject as closer neighbor than a time series sample when comparing the relative taxonomic abundances. For example, the d_392_ sample from *Scavenger* has a sample from another subject as fifth neighbor instead of the *Scavenger* d_0_ sample. The number of samples having another subject as closer neighbor rather than a time series sample from the same subject increased when clustering was performed using relative COG and KO abundances (Additional files 2 and 3).

To characterize the temporal variability of the community structure, we calculated the ecological indices, such as the Shannon diversity index, and found that they varied little over time for all subjects (Figure 5A, lower panel for diversity) except *Alien*, who underwent antibiotics treatment.

Our results support previous studies reporting that the temporal variability of the species composition within a subject is smaller than between-subject variability and that in the absence of larger perturbation each individual’s microbiota remains relatively stable over time [6,8,9,11,12,14,24,26-29]. However, here we show that even in the context of a large cohort of fecal metagenomes the subjects can be resolved based on the taxonomic composition of their fecal metagenomes with very few exceptions. Thus, the gut microbiome, if unperturbed, is highly subject-specific and the variability is small compared to the between-subject variability.

### The effect of perturbations on fecal microbial communities

During the time period of the study, one subject (*Alien*) suffered from an infection that was treated with antibiotics and underwent colonoscopy screening, which required bowel cleanse. The antibiotics treatment comprised four days with ceftriaxone, a third-generation cephalosporin antibiotic with broad-spectrum activity against Gram-positive and Gram-negative bacteria. To investigate the consequences of these medical treatments, additional samples were collected for this subject after antibiotics intake (d_376_, d_377_ and d_380_) and bowel cleanse (d_630_, d_632_, and d_637_) (Figure 1, sub-studies 4 and 5; for further details see Material and methods).

To observe the response of the fecal microbiome to these two perturbations, we performed hierarchical clustering and found that the post-antibiotics samples separated from the pre-antibiotics samples, but were still distinct from other subjects (Figure 5A,B). The samples taken 226 to 399 days after antibiotics treatment (d_600–773_) clustered closer to the pre-treatment samples (Figure 5A,B), suggesting a (partial) recovery. Determining the nearest neighbor samples in the context of the 888 HMP and MetaHIT samples, using Euclidean distance on taxonomic and functional profiles, confirmed the aforementioned observation suggesting that the gut community composition was still distinct even 399 days (d_773_) post-antibiotics treatment but gained similarity with the pre-antibiotics community composition (Figure 4). A similar pattern was observed for nearest-neighbor analysis of COG abundances, but individual specificity was less clear for KO abundances (Additional files 2 and 3).

The immediate post-treatment samples (d_376–380_) showed a drastic reduction in Shannon diversity index, species richness and evenness, indicating that fewer and less evenly abundant microbial species were detected. The Shannon diversity index, species richness and evenness of the post-treatment samples dropped from 3.5 to 0.2, 100 to 37 and 0.75 to 0.05, respectively, and were still reduced at d_392_ (18 days after the treatment), compared to the pre-treatment state. At d_600_, the diversity had returned to its initial level (Figure 5A, lower panel), yet the samples still clustered separately from the pre-treatment samples, indicating that the recovery is not complete (Figures 4 and 5B). Separation of community profiles from the initial state along the first principal coordinate in an ordination analysis (using Jensen-Shannon divergence) correlated with the decline of the Shannon diversity index (Pearson correlation rho = 0.86, *P* = 3.9e^−14^; Figure 5A).

The bowel cleanse on the day before d_630_ did not have a considerable effect on the community composition: the samples cluster closely with d_600_, which was before colonoscopy and the fluctuation of the Shannon diversity index was similar to the other subjects and notably smaller than the impact of the antibiotics treatment (Figure 5A,B). Although this case study comprises only one subject, our result that bowel cleanse has little effect on gut microbiome composition is in line with the finding by O’Brien *et al*. [43].

It has been reported that antibiotics have a strong impact on the gut microbial community composition for an extended period of time, although the community was sometimes found to be similar to its pretreatment state within weeks. The return was subject-dependent and often incomplete, at least for some species monitored for time periods of two to six months [14,17] and up to two years [19,44]. Even though we studied the effect of antibiotics in only one subject, we can show that, at least in this subject, despite species diversity recovery, the gut microbial composition was still distinct from the pre-treatment state, even 399 days after the antibiotics treatment. It would be worthwhile exploring in the future how antibiotics effects vary between subjects and depends on factors such as dosage, duration of the treatment and type of antibiotics.

## Conclusion

Several studies have addressed the temporal variability or the technical variability (for example, induced by different DNA isolation methods or preservation techniques) of the gut microbiome separately but none set them in a broader context. Hence, these studies have so far not disentangled the biological temporal variability (like, for example, community shifts due to disease or medication) from technical variability (for example, induced by preservation conditions or insufficient stool homogeneity).

In our study we provide the to date largest metagenomic data set of fecal samples collected over more than two years. We addressed the aspects of comparing technical and temporal variability, finding that temporal variability within each subject’s gut microbiome was smaller than that between subjects. Even in the context of 888 metagenomes, all time series samples could be recovered using taxonomic abundances, as long as antibiotics did not perturb the gut microbiome. The technical variability introduced by RNALater was small compared to freezing, for both taxonomic and functional features, and does not disrupt subject-specificity nor time point-specificity of the gut microbiome. Thus, we suggest RNALater as an alternative to freezing for the preservation of the fecal microbiome for metagenomic studies.

## Material and methods

### Sample collection

#### Fecal sample collection for time series

Informed consent to obtain time series samples of fecal samples was obtained from seven healthy subjects in Germany through the my.microbes project [45]. The study protocol was approved by the EMBL Bioethics Internal Advisory Board, and is in agreement with the WMA Declaration of Helsinki. All subjects were living in Heidelberg, Germany at the beginning of the study and the mean age of the subjects upon enrollment was 34 ± 6 years. Among these subjects were five males (*Alien*, *Bugkiller*, *Peacemaker*, *Halbarad* and *Scavenger*) and two females (*Daisy* and *Tigress*). Subjects reported themselves as healthy, if they did not undergo prescribed medical treatment or showed any indication of disease symptoms. Fecal samples were collected and conserved under anaerobic conditions in a sealed bag, kept at −20°C for short-term storage and stored at −80°C upon arrival in the laboratory. The fecal samples were collected at days 0, 2, 7, 60, 392, 600 and 773 (sub-study 3) and are referred to here as d_0_, d_2_, d_7_ and so on. One male subject (*Alien*) contracted a bacterial infection and collected further samples after being hospitalized and receiving 2 g of ceftriaxone. Ceftriaxone is an antibiotic with broad-spectrum activity against Gram-positive and Gram-negative bacteria that was administered parenterally over 4 days. The last injection was two days before the first sampling time point (d_376_) and further samples were then collected on the subsequent days (d_377_, d_378_ and d_380_; sub-study 4). Additional samples were taken starting one day after undergoing bowel cleanse for routine colonoscopy (d_630_, d_632_ and d_637_; sub-study 5). Figure 1 shows the study design in detail and metadata and sequencing information are given in the Additional file 1.

#### Fecal sample collection for method comparison

In parallel to the fresh frozen fecal samples, additional samples (1 g each) were collected from five subjects at time point d_7_ and from three subjects at d_392_ (without homogenization) and were stored in 10 ml RNALater® Stabilization Solution (Life Technologies GmbH, Darmstadt, Germany). Short-term storage was either at +4 to 10°C or at RT for one week (d_7_, sub-study 1) or at +4°C (d_392_, sub-study 2) for 24 h and frozen at −80°C upon arrival in the laboratory. At d_392_, each subject collected samples in triplicate, preserved in both RNALater and freshly frozen (Figure 1).

#### Inclusion of published fecal metagenomes

Published metagenomes from MetaHIT [2,46,47] and HMP [1] were included in our study to set our time series in context of a large collection of metagenomes.

### Sample processing and sequencing

#### DNA isolation from fecal samples

One milliliter of defrosted samples immersed in RNALater was taken and diluted with sterile phosphate-buffered saline and pelleted by centrifugation. Genomic DNA was extracted from frozen or RNALater-preserved fecal samples as previously described [48] using the G’NOMEs kit (MP Biomedicals, Illkirch, France). The following minor modifications were made to the protocol: cell lysis/denaturation was performed (30 minutes, 55°C) before protease digestion was carried out overnight (55°C). Mechanical lysis was followed by RNAse digestion (50 μl, 30 minutes, 55°C). The purified DNA was resuspended in TE buffer after final precipitation for storage at −20°C.

#### Library preparation and metagenomic sequencing

Library generation and whole genome shotgun sequencing of the fecal samples was carried out on the Illumina HiSeq 2000/2500 (Illumina, San Diego, CA, USA) platform as described in Zeller *et al*. [49]. All samples were paired-end sequenced with 100 bp read lengths at the Genomics Core Facility, European Molecular Biology Laboratory, Heidelberg, to a sequencing depth of approximately 5 Gbp (see Additional file 1 for sequencing results).

### Data processing

#### Taxonomic profiling of fecal samples

Using MOCAT [50], a software package used to process raw Illumina reads to generate taxonomic and functional profiles (option screen with alignment length cutoff 45 and minimum 97% sequence identity), taxonomic relative abundance profiles were generated by mapping screened HQ reads from each metagenome to a database consisting of 10 universal single-copy marker genes extracted from 3,496 NCBI reference genomes and 263 human gut metagenomes that had previously been clustered and linked by co-variance into mOTUs [29,51]. Quantification of mOTU linkage groups was performed using MOCAT, but is also available as a standalone tool at [29].

#### Functional profiling of fecal samples

Using MOCAT [50] (option screen with alignment length cutoff 45 and minimum 95% sequence identity) functional relative abundance profiles were generated by first calculating gene abundance profiles by mapping screened HQ (high quality) reads from each metagenome to an functionally annotated database consisting of predicted genes from 263 human gut metagenomes [29,49], and estimating each gene’s abundance as gene length-normalized nucleotide counts of all reads that matched the protein-coding region of the gene. And second, for each functional feature, its abundance in the metagenomic gene pool was estimated as the sum of the relative abundances of all genes belonging to this family. The genes were summarized into COGs [40], and KEGG KOs, modules and pathways [41]. The metagenomic gene catalog had already been functionally annotated to the KEGG database [48], and was additionally annotated to different COGs by aligning the translated amino acid sequence of each gene to the eggNOG (version 3) [40] database using BLAST (version 2.2.24) [52] (maximum e-value 0.01) and then annotating the genes using SmashCommunity (version 1.6) [53].

### Data analysis

For the statistical data analysis at the species level, mOTU abundances were used [29] and samples were included in the data analysis if they had more than 3,800 insert counts.

#### Ecological indices

For the comparison of RNALater with fresh frozen feces and time series samples with each other, mOTU abundances [29] were used to calculate Shannon diversity index, evenness and species richness. To standardize sampling depth, richness, Shannon diversity index and evenness were assessed after rarefaction of the insert count tables to 3,800 insert counts per sample. Differences were assessed using the Wilcoxon test (unpaired) on the deviation from the mean of each subject. *P*-values ≤0.05 were considered statistically significant.

#### Clustering

Principal coordinate analyses and complete linkage clustering of Euclidean distances (Figure 2A) and Jensen-Shannon divergence distances (Figure 5A) were performed using the *ape* and *ade4* R packages. The dendrograms shown are based on Euclidean distance measurements on the logged abundances (Figures 2B and 3B,C). Nearest neighbors were determined to be the samples with the smallest Euclidean distance (Figure 4; Additional files 2 and 3). Due to large differences in sequencing depth, the metagenomes collected in this study and the HMP [1] and MetaHIT [2,46,47] taxonomic data were only analyzed after rarefaction to an insert count of 5,000 per sample. All samples that passed these criteria were included in the functional analysis.

#### Two-way ANOVA

To observe taxonomic and functional-specific biases introduced by RNALater preservation across all subjects, a two-way ANOVA was performed for d_7_ and d_392_ separately. Our setup was analogous to a previous analysis [36]. Relative species, COG, KO, module and pathway abundances were arcsine square root transformed (for variance stabilization) and only features with a relative abundance of more than 0.01% in at least three samples were included. For d_392_, the median value of the three replicates for each feature was used.

### Data availability

The shotgun metagenomic sequencing data from this study are available from the European Nucleotide Archive (ENA) database [54], accession number ERP009422.

### Description of additional data files

The following additional data are available with the online version of this paper. Additional file 1 is a table listing the metadata and sequencing information of the analyzed samples. Additional file 5 is a table listing statistically significant taxonomic and functional features resulting from the method comparison.

## Additional files

Additional file 1: Table S1. Overview of the metadata of the subjects and sequencing information of the samples included. Additional file 2: Figure S1. Nearest neighbor plot based on COGs. Additional file 3: Figure S2. Nearest neighbor plot based on KOs. Additional file 4: Figure S3. Comparison of technical, temporal and between-subject variability based on functional profiles. Additional file 5: Table S2. Overview of taxonomic and functional features. The features include those above the horizontal black line in Figure 3 and Additional file 4.

## Abbreviations

COG: cluster of orthologous groups
HMP: Human Microbiome Project
KEGG: Kyoto Encyclopedia of Genes and Genomes
KO: KEGG group of orthologous genes
MetaHIT: Metagenomics of the Human Intestinal Tract
mOTU: metagenomic operational taxonomic unit
PCR: polymerase chain reaction
RT: room temperature
