# Supplementary figures and images for: Temporal and technical variability of human gut metagenomes
Source: Genome Biol. 2015 Apr 8;16(1):73. doi: 10.1186/s13059-015-0639-8 (PMC4416267; doi:10.1186/s13059-015-0639-8)

Nearest neighbor plot (COGs)

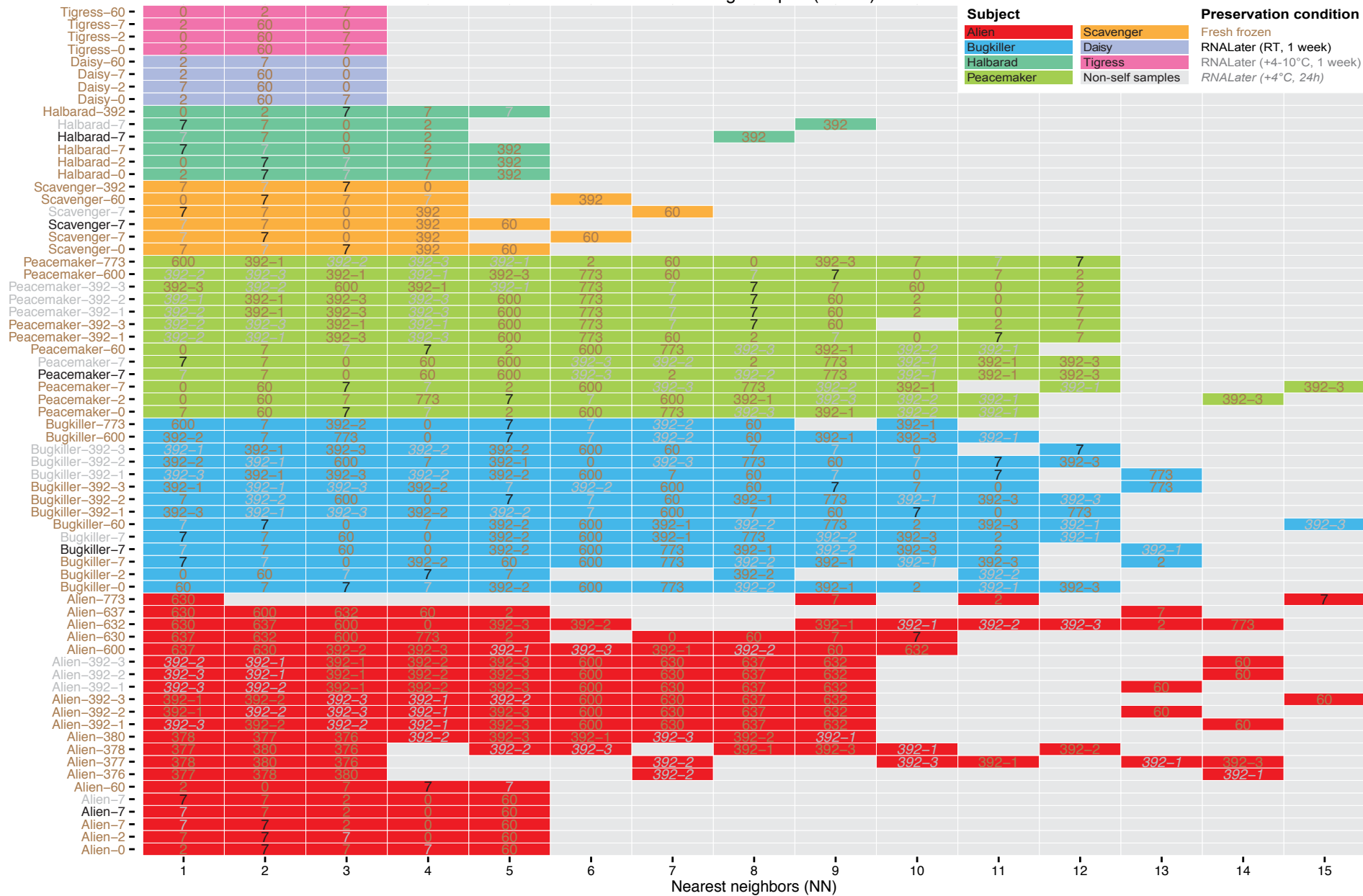

Supplement: Additional file 2: Figure S1. — Nearest neighbor plot based on COGs. [file 13059_2015_639_MOESM2_ESM.pdf]

Nearest neighbor plot (KEGG KOs)

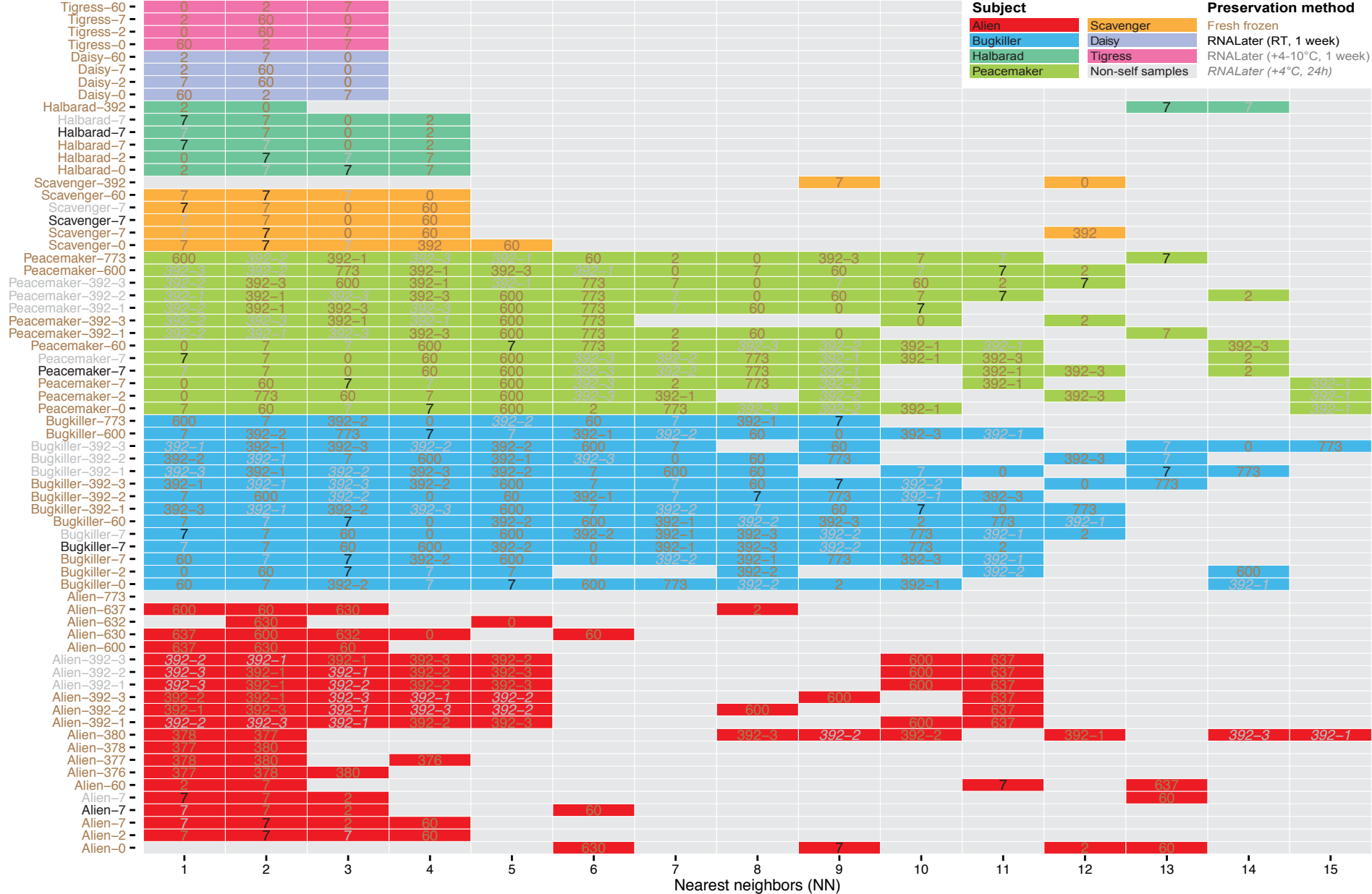

Nearest neighbors (NN)

Supplement: Additional file 3: Figure S2. — Nearest neighbor plot based on KOs. [file 13059_2015_639_MOESM3_ESM.pdf]

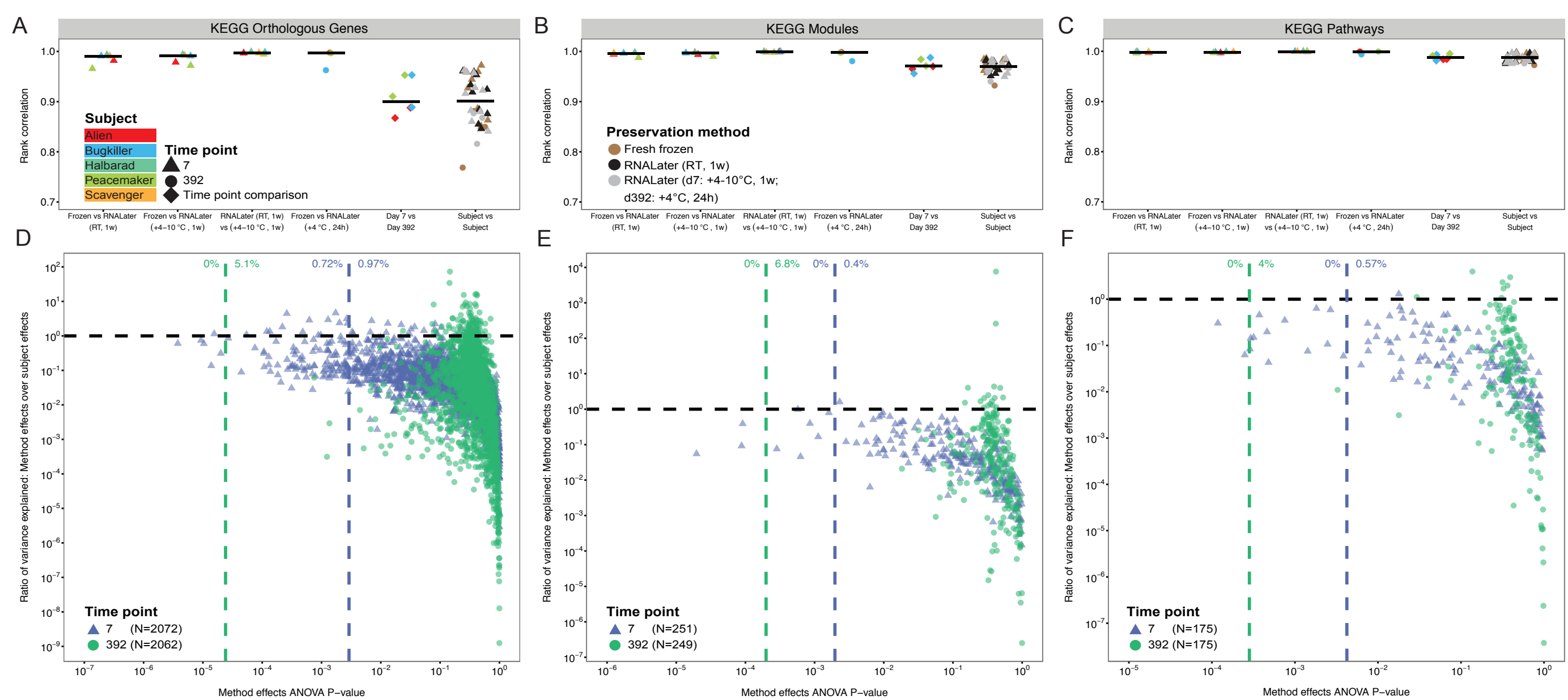

Supplement: Additional file 4: Figure S3. — Comparison of technical, temporal and between-subject variability based on functional profiles. [file 13059_2015_639_MOESM4_ESM.pdf]
